# Differences in the Intracellular Localization of Methylated β-Cyclodextrins-Threaded Polyrotaxanes Lead to Different Cellular States

**DOI:** 10.3390/biom13060903

**Published:** 2023-05-29

**Authors:** Yuma Yamada, Shinnosuke Daikuhara, Atsushi Tamura, Kei Nishida, Nobuhiko Yui, Hideyoshi Harashima

**Affiliations:** 1 Faculty of Pharmaceutical Sciences, Hokkaido University, Sapporo 060-0812, Japan; 2 Fusion Oriented Research for Disruptive Science and Technology (FOREST) Program, Japan Science and Technology Agency (JST), Tokyo 102-8666, Japan; 3 Institute of Biomaterials and Bioengineering, Tokyo Medical and Dental University, Tokyo 101-0062, Japan

**Keywords:** autophagy, polyrotaxane, mitochondria, endoplasmic reticulum, MITO-Porter, cell biology

## Abstract

Activation of autophagy represents a potential therapeutic strategy for the treatment of diseases that are caused by the accumulation of defective proteins and the formation of abnormal organelles. Methylated β-cyclodextrins-threaded polyrotaxane (Me-PRX), a supramolecular structured polymer, induces autophagy by interacting with the endoplasmic reticulum. We previously reported on the successful activation of mitochondria-targeted autophagy by delivering Me-RRX to mitochondria using a MITO-Porter, a mitochondria-targeted nanocarrier. The same level of autophagy induction was achieved at one-twentieth the dosage for the MITO-Porter (Me-PRX) compared to the naked Me-PRX. We report herein on the quantitative evaluation of the intracellular organelle localization of both naked Me-PRX and the MITO-Porter (Me-PRX). Mitochondria, endoplasmic reticulum and lysosomes were selected as target organelles because they would be involved in autophagy induction. In addition, organelle injury and cell viability assays were performed. The results showed that the naked Me-PRX and the MITO-Porter (Me-PRX) were localized in different intracellular organelles, and organelle injury was different, depending on the route of administration, indicating that different organelles contribute to autophagy induction. These findings indicate that the organelle to which the autophagy-inducing molecules are delivered plays an important role in the level of induction of autophagy.

## 1. Introduction

Autophagy is a cellular degradation system that degrades and reuses intracellular proteins and organelles [1,2,3]. This system plays a role in the quality control of cellular components, and its dysfunction has been reported to be associated with the onset of a number of diseases [4,5]. Therefore, artificially inducing autophagy and enhancing cell metabolism would be expected to be an innovative therapeutic strategy for the treatment of various diseases that are difficult to treat.

We previously reported on the design of methylated β-cyclodextrins (Me-β-CDs)-threaded acid-labile polyrotaxane (Me-PRX) that induces autophagy [6]. The Me-β-CDs released from the Me-PRX would be predicted to interact with organelle membranes and induce autophagy through the leakage of cholesterol from the membrane. The naked Me-PRX was observed to co-localize with the endoplasmic reticulum (ER) and lysosomes, and autophagy was induced by ER stress [6].

In addition, we successfully activated mitochondria-targeted autophagy by the delivery of Me-RRX to mitochondria [7]. A MITO-Porter, a mitochondria-targeted nanocarrier [8,9,10,11], was used for the delivery of cargoes to mitochondria. In this experiment, we packaged Me-PRX in the MITO-Porter to prepare an S2-MITO-Porter (Me-PRX) that was modified with the S2 peptide (S2, Dmt-d-Arg-F-K-Dmt-d-Arg-F-K, Dmt = 2, 6-dimethyltyrosine), which has cellular uptake and mitochondrial targeting activity [12]. When the same amount of Me-PRX was internalized into cells that had been treated with the S2-MITO-Porter (Me-PRX) and naked Me-PRX, respectively, a large difference was observed in the level of induction of autophagy. The S2-MITO-Porter (Me-PRX) induced autophagy much more strongly compared to the naked PRX. These findings indicate that a difference in the subcellular localization of an autophagy-inducing molecule such as Me-PRX affects the level of induction of autophagy.

**Figure 1:**
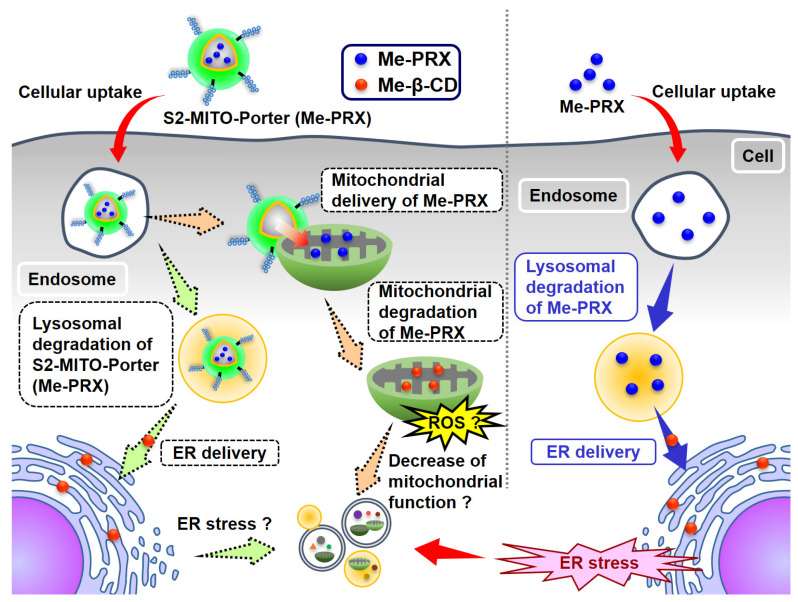
Schematic diagram illustrating autophagy induction mechanism by S2-MITO-Porter (Me-PRX).

The predicted intracellular fate of Me-PRX and the S2-MITO-Porter (Me-PRX) to induce autophagy is shown in Figure 1. To validate this hypothesis, we first evaluated the quantitative intracellular organelle localization of Me-PRX and the S2-MITO-Porter (Me-PRX) using confocal laser scanning microscopy (CLSM). Mitochondria, ER and lysosomes were selected as target organelles because they would be involved in autophagy induction. In addition, organelle injuries were evaluated by measuring mitochondrial membrane potentials and detecting the C/EBP homologous protein (CHOP), which is an ER stress-inducible protein that is involved in the regulation of programmed cell death [13]. Finally, cell viability was also evaluated using a WST-1 assay. The findings of this study indicate that the specific organelle in which the autophagy-inducing molecules are localized is an important determinant of the level of activation of the induced autophagy.

## 2. Materials and Methods

### 2.1. Materials

1,2-Dioleoyl-sn-glycero-3-phosphatidyl ethanolamine (DOPE) was purchased from the NOF Corporation (Tokyo, Japan). Sphingomyelin (SM) was purchased from Avanti Polar Lipids (Alabaster, AL, USA). STR-S2 (stearylated-Dmt-d-Arg-FK-Dmt-d-Arg-FK-NH_2_, Dmt = 2,6-dimethyltyrosne) was obtained from the Toray Research Center (Tokyo, Japan). The synthesis of methylated β-cyclodextrin-threaded acid-labile PRX (Me-PRX) and BODIPY-labeled Me-PRX [14] are described in the Supplementary Materials. HeLa human cervix carcinoma cells were obtained from the RIKEN Cell Bank (Tsukuba, Japan). Dulbecco’s modified Eagle’s medium (low glucose) (DMEM) was obtained from Wako Pure Chemical Industries, Ltd. (Osaka, Japan). Fetal bovine serum (FBS) was obtained from Sigma–Aldrich (St. Louis, MO, USA). CellLight ER-RFP BacMam 2.0, LysoTracker Red DND-99 and MitoTracker Deep Red FM were obtained from Thermo Fisher Scientific (Waltham, MA, USA). All other chemicals used were commercially available reagent-grade products.

### 2.2. Construction of the S2-MITO-Porter (Me-PRX)

Chloroform (500 µL) containing 2.75 µmol lipids [DOPE/SM = 9:2 (molar ratio)] and 500 µL of diisopropyl ether were added to a glass tube. Additionally, 500 µL of 10 mM HEPES buffer (pH 7.4) containing Me-PRX (4 mM, final concentration of Me-β-CD) was added, followed by sonication for 15 s with a probe-type sonicator (SONIFIER Model 250D, BRANDSON, Kanagawa, Japan). After removing the organic solvent with a stream of N_2_, a liposome suspension was formed by sonication for 30 sec in a bath-type sonicator (AU-25C, Aiwa Company, Tokyo, Japan). Finally, the STR-S2 solutions (10 mol% of total lipids) were added to the liposome suspension to produce the S2-MITO-Porter (Me-PRX). To investigate the intracellular trafficking of Me-PRX, an S2-MITO-Porter (Me-PRX) containing fluorescently labelled Me-PRX (BODIPY-Me-PRX) was used.

### 2.3. Intracellular Observation of BODIPY-Me-PRX in Cells Stained with Mitochondria or Lysosomes Using CLSM

HeLa cells (4 × 10^4^ cells/dish) were incubated on a 3.5 cm glass-base dish (Iwaki) for 48 h (5% CO_2_, 37 °C). First, the DMEM (FBS+) containing naked BODIPY-Me-PRX [green color] (1000 μM, final concentration of β-CD) or S2 MITO-Porter (Me-PRX) containing BODIPY-Me-PRX (40 μM, final concentration of Me-β-CD) was added to the cells after washing the cells in DMEM (FBS+), followed by incubation for 16 h (5% CO_2_, 37 °C). Next, the medium was replaced with fresh DMEM (FBS+) containing MitoTracker Deep Red [red color] (60 nM, final concentration) or LysoTracker Red DND-99 [red color] (1 μM, final concentration) and the cells were then incubated for 20 min (5% CO_2_, 37 °C). After washing the cells in Phenol red-free DMEM (FBS+), they were observed by CLSM (FV10i-LIV). The details of CLSM settings are described in the Supplementary Materials. 

### 2.4. ER Staining and the Intracellular Observation of BODIPY-Me-PRX Using CLSM

HeLa cells (4 × 10^4^ cells/dish) were incubated on a 3.5 cm glass-base dish for 24 h (5% CO_2_, 37 °C). After washing the cells in DMEM (FBS+), the DMEM (FBS+) containing CellLight ER-RFP BacMam 2.0 was added to the cells to stain the ER red, followed by incubation for 24 h (5% CO_2_, 37 °C). The DMEM (FBS+) containing naked BODIPY-Me-PRX [green color] (1000 μM, final concentration of Me-β-CD) or S2 MITO-Porter (Me-PRX) containing BODIPY-Me-PRX (40 μM, final concentration of Me-β-CD) was added to the cells after washing the cells in DMEM (FBS+), followed by incubation for 16 h (5% CO_2_, 37 °C). After washing the cells in Phenol red-free DMEM (FBS+), they were observed by CLSM (FV10i-LIV). The details of the CLSM setting are described in the Supplementary Materials.

### 2.5. Evaluation of the Occupancy Rates of Mitochondria, ER and Lysosome

The occupancy rates of mitochondria, ER and lysosome were calculated based on the CLSM images of stained images, as described below. The occupancy rates for Me-RPX for mitochondria, ER and lysosomes were evaluated using Image Pro-Plus 7.0 (Ropper Industries, Sarasota, FL, USA). Fluorescent and bright-field cell images were obtained after samples were stained with BODIPY labeled Me-PRX (green), followed by staining mitochondria, ER and lysosomes. These organelles are indicated in red color and were captured by means of CLSM. Each eight-bit TIFF image was analyzed to quantify the total area of each region of interest. The yellow pixel areas where carriers (green) were co-localized with stained organelle (red) are marked in each image. The yellow and red colored pixel areas of each cluster in the cell, s_i_ (yellow) and s_i_ (red), were separately summed for each image and are denoted as S′_z=j_ (yellow) and S′_z=j_ (red), respectively. The values of S′_z=j_ (yellow) and S′_z=j_ (red) in each image were further summed and are denoted as S (yellow) and S (red), respectively. S (yellow) and S (red) represent the total area of carriers that were co-localized with the organelle of interest and include all of the Me-RPX inside the cell and the organelle region in the total cell. The occupancy rates of mitochondria, ER and lysosome were calculated as follows

Organelle occupancy rate (%) = S (yellow)/S (red) × 100

**Figure 2:**
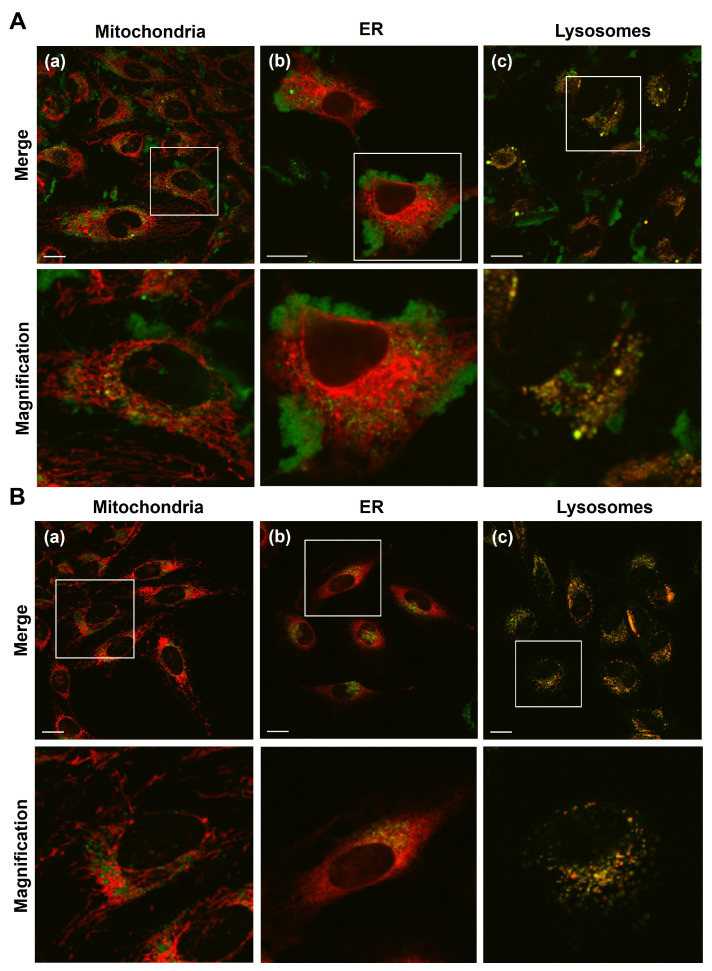

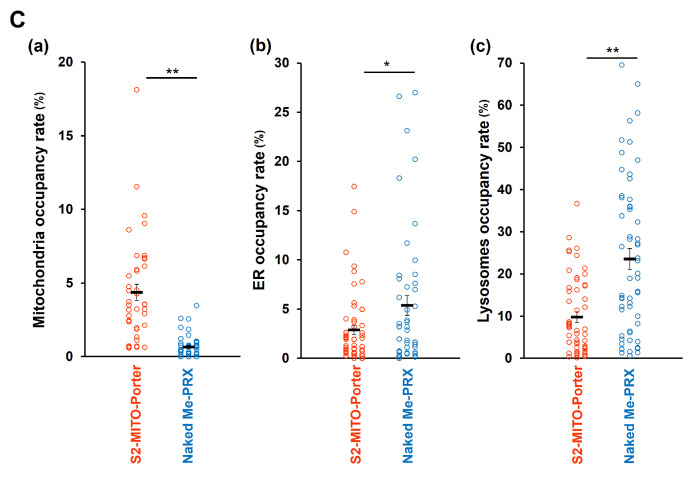
Intracellular observation of the Me-PRX and evaluation of organelle occupancy. HeLa cells were incubated with the BODIPY (a fluorescence dye)-labeled Me-PRX encapsulated in the S2-modified MITO-Porter (final conc. of Me-β-CD, 40 µM) (**A**) and naked BODIPY-labeled Me-PRX (final conc. of Me-β-CD, 1000 µM) (**B**) for 16 h. After staining the organelle, the cells were observed by CLSM. The green color indicates BODIPY-labeled Me-PRX (**A**,**B**; (**a**–**c**)). The red color indicates mitochondria (**a**), ER (**b**) and lysosome (**c**), respectively. BODIPY-labeled MITO-Porter appeared as yellow clusters when it was localized in the organelle (mitochondria, ER or lysosome)—scale bars; 20 μm. In (**C**), the occupancy rates of mitochondria (**a**), ER (**b**) and lysosome (**c**) were calculated based on the CLSM images shown in (**A**,**B**) (see Section 2 for the details). Organelle occupancy that the carriers accumulated within the organelle region of the whole organelle region in the cell are summarized. Circles represent the values of individual cells summarized in each treatment. Bars are the mean value (n = 41–58). Significant differences were calculated by Student’s *t*-test (** *p* < 0.01, * *p* < 0.05).

The organelle occupancy that the carriers accumulated within the region of the whole organelle region in the cell are summarized in Figure 2C and Figure S1.

### 2.6. Evaluation of Mitochondrial Membrane Potential

Samples were incubated with HeLa cells (1 × 10^5^ cells/well) seeded on 6-well plates in 1 mL of serum containing DMEM for 24 h under an atmosphere of 5% CO_2_/air at 37 °C. The cells were stained with Tetramethylrhodamine, methyl ester (TMRM) (final concentration, 1 μM) for 20 min and then washed with phosphate-buffered saline (PBS (-)), trypsinized, suspended in DMEM with serum, and precipitated by centrifugation (700× *g*, 4 °C, 3 min). After washing the cells with FACS buffer (0.5% bovine serum albumin in PBS (-)), the cells were resuspended in FACS buffer, and the fluorescence of TMRM was analyzed by flow cytometry (FACS Gallios; Beckman Coulter, Brea, CA, USA).

### 2.7. Detection of CHOP Expression by Western-Blotting

HeLa cells (5 × 10^4^ cells/well) were incubated on a 12-well plate (Thermo Fisher Scientific Inc., Waltham, MA, USA) for 24 h (5% CO_2_, 37 °C). After washing the cells with PBS (-), the DMEM (FBS+) containing samples was added to the cell suspension, followed by incubation for 16 h (5% CO_2_, 37 °C). After washing the cells with PBS (-), the cells were lysed with RIPA buffer containing protease and phosphatase inhibitors. The lysate was centrifuged (20,630× *g*, 10 min, 4 °C), and the supernatant was collected, followed by western blotting (see Supplementary Information for the details).

### 2.8. Evaluation of Cell Toxicities

Cell viability was measured using the WST-1 assay. The cells were cultured on a 24-well plate (Corning Inc., Corning, NY, USA) for 24 h under an atmosphere of 5% CO_2_/air at 37 °C. The sample was transfected into the cells in DMEM (FBS+) and incubated for 48 h. The WST-1 reagent was added to the cells immediately after irradiation and then incubated for 2 h. The change in reagent absorbance was measured at 450 nm with the reference at 630 nm using a microplate photometer (EnSpire^®^ Multimode Plate Reader, Perkin Elmer; Waltham, MA, USA).

### 2.9. Observation of Cell Form Using Microscopy

HeLa cells (1 × 10^5^ cells/well) were seeded on 6-well plates in one day before being assayed and incubated in DMEM that contained 10% FBS under an atmosphere of 5% CO_2_/air at 37 °C. The cells were washed with PBS (-) and then incubated with carriers containing Me-PRX or naked Me-PRX suspended in the medium for 24 h under an atmosphere of 5% CO_2_/air at 37 °C. The cells were observed by microscopy.

### 2.10. Statistical Analysis

Data are expressed as the mean ± S.D. for the indicated number of experiments. For multiple comparisons, one-way ANOVA followed by the Student Newman Keuls (SNK) test was performed. Levels of *p* < 0.05 were considered to be significant.

## 3. Results

### 3.1. Intracellular Observation of Me-PRX

To analyze changes in the intracellular localization of Me-PRX, BODIPY (a green fluorescence dye)-labeled Me-PRX encapsulated S2-modified MITO-Porter and naked BODIPY-labeled Me-PRX (S2-MITO-Porter (Me-PRX)) were added to HeLa cells, and the accumulation of Me-PRX in mitochondria, the ER and lysosomes were observed (Figure 2A,B). Mitochondria stained with MitoTracker Deep Red and lysosomes stained with LysoTracker Red DND-99 are displayed in a pseudo color as red. To detect ER, ER-RFP-expressing HeLa cells showing the ER as red were used. The results indicated that Me-PRX was taken by the cells and was co-localized with lysosomes in the case of both the S2-MITO-Porter (Me-PRX) and naked Me-PRX. In the case of the S2-MITO-Porter (Me-PRX), the Me-PRXs were largely co-localized with mitochondria compared to the ER (Figure 2A), whereas naked Me-PRXs were co-localized largely with the ER, with only traces being localized in mitochondria (Figure 2B).

To perform a quantitative assessment, the organelle occupancy of Me-PRX was calculated as a percentage of the organelle area occupied by the localized Me-RPX using image analysis software (Figure 2C and Figure S1A,B). The results showed that the Me-PRX that was introduced into cells by the S2-MITO-Porter (Me-PRX) accumulated not only in mitochondria and lysosomes but also in the ER (Figure S1A). Naked Me-PRX was found to accumulate largely in the ER and lysosomes, while the accumulation in mitochondria was very low (Figure S1B). A comparison of organelle occupancy between S2-MITO-Porter (Me-PRX) and naked Me-PRX (Figure 2C) showed that the mitochondrial occupancy of Me-PRX was significantly increased by using the S2-MITO-Porter (Me-PRX), substantially increasing the mitochondrial delivery of Me-PRX. In addition, compared to naked Me-PRX, the ER occupancy and lysosomal occupancy of Me-PRX were reduced when the S2-MITO-Porter (Me-PRX) was used.

### 3.2. Evaluation of Mitochondrial Membrane Potentials

To validate the effect of the S2-MITO-Porter (Me-PRX) on intracellular mitochondrial function, mitochondrial membrane potential was evaluated. TMRM, a mitochondrial membrane potential-dependent staining reagent used for the evaluation, accumulates at high concentrations and fluoresces in mitochondria with normal membrane potential, whereas when the mitochondrial membrane potential is reduced, it diffuses, and its fluorescence intensity decreases.

**Figure 3:**
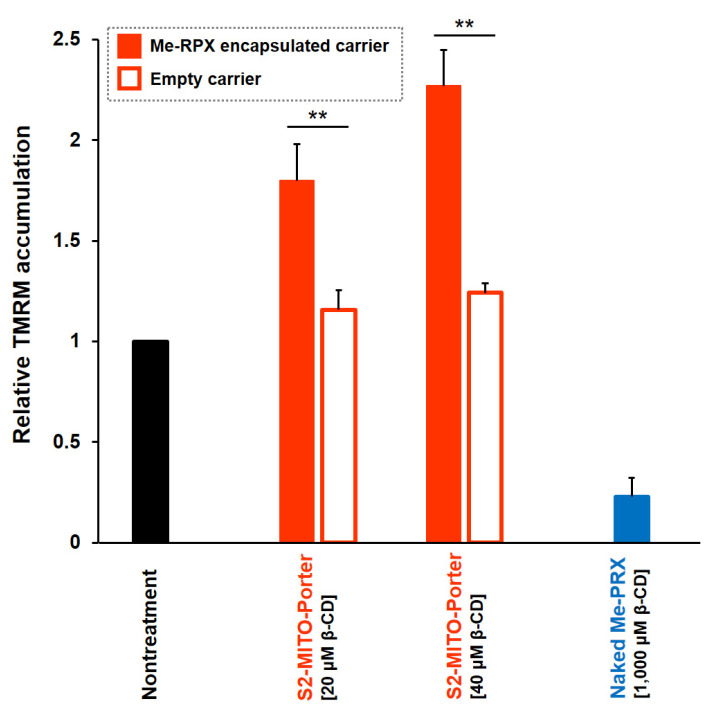
Evaluation of mitochondrial membrane potentials. At 24 h after the addition of S2-MITO-Porter (Me-PRX) (final conc. of Me-β-CD, 20, 40 µM), the empty S2-MITO-Porter (lipid concentration equivalent to the S2-MITO-Porter (Me-PRX)) and naked Me-PRX (final conc. of Me-β-CD, 1000 µM) to HeLa cells, mitochondria were stained with TMRM, and TMRM fluorescence was measured using a flow cytometer. The staining of mitochondria with TMRM is dependent on the membrane potential; therefore, TMRM cannot stain mitochondria when the membrane potential is lost. The relative accumulation of TMRM was calculated as a relative value obtained by dividing the TMRM fluorescence of the sample-treated group by the TMRM fluorescence of the untreated cell group. Data are represented as the mean ± S.D. (n = 3). Significant differences were calculated by Student’s *t*-test (** *p* < 0.01).

Twenty-four hours after the addition of the S2-MITO-Porter (Me-PRX) to HeLa cells, TMRM fluorescence was measured using a flow cytometer and the relative mitochondrial membrane potential was calculated (Figure 3). The results showed that using the S2-MITO-Porter (Me-PRX) at 20 and 40 µM, Me-β-CD significantly increased mitochondrial membrane potential. Conversely, a decrease in mitochondrial membrane potential was observed in the case of naked Me-PRX at 1000 µM β-CD.

### 3.3. Quantification of CHOP Expression after Activation with the S2-MITO-Porter (Me-PRX)

Me-PRX is taken up by cells and accumulates in the ER, where it induces autophagy by inducing ER stress [6]. In such a condition, the intracellular reticular structure of the ER changes to a dot-like shape and its expression is induced by endoplasmic reticulum stress and the nuclear translocation and expression of the C/EBP homologous protein (CHOP), a transcription factor whose expression is induced by ER stress, would be predicted to increase [13]. Since Me-PRX was observed to be translocated not only to mitochondria but also to the ER when S2-MITO-Porter (Me-PRX) was used (Figure 2A,C and Figure S1A), we considered the possibility that ER stress was induced in the same way when the S2-MITO-Porter (Me-PRX) was used.

**Figure 4:**
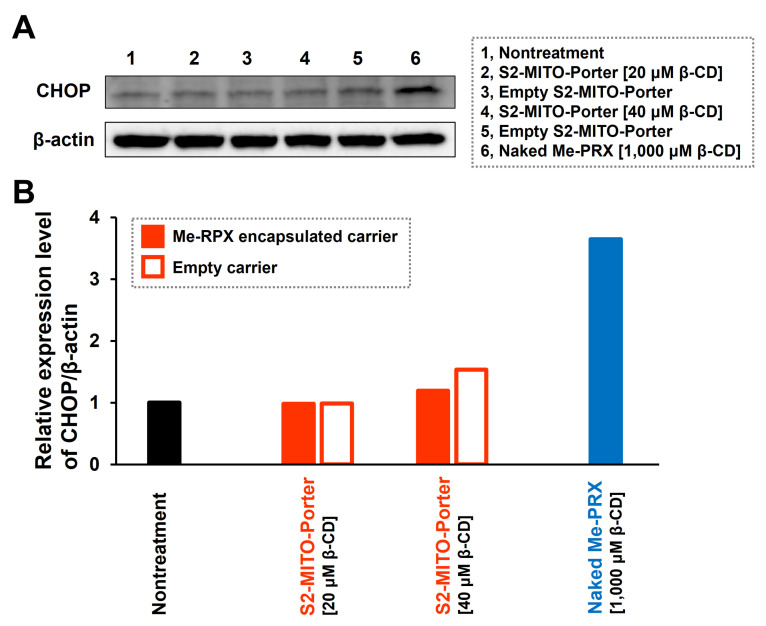
Quantification of ER stress marker CHOP expression. At 24 h after the addition of S2-MITO-Porter (Me-PRX) (final conc. of Me-β-CD, 20, 40 µM), empty S2-MITO-Porter (lipid concentration equivalent to S2-MITO-Porter (Me-PRX)) and naked Me-PRX (final conc. of Me-β-CD, 1000 µM) to HeLa cells, level of expression of CHOP was evaluated by Western blotting (**A**). The band intensities of CHOP and β-actin obtained by Western blotting were quantified by image analysis software, and the relative expression level of CHOP/β-actin was calculated with untreated cells as 1 (**B**).

To verify the effect of the S2-MITO-Porter (Me-PRX) on ER function, the S2-MITO-Porter (Me-PRX) was added to HeLa cells, and the level of expression of CHOP was assessed by Western blotting 24 h after the treatment (Figure 4). The results showed an increase in CHOP expression in the case of Me-PRX alone (1000 µM Me-β-CD), confirming the induction of ER stress. On the other hand, no change in CHOP expression was observed in the case of the S2-MITO-Porter (Me-PRX) (20, 40 µM Me-β-CD), indicating no induction of ER stress.

### 3.4. Evaluation of Cell Viability after Treatment with S2-MITO-Porter (Me-PRX)

**Figure 5:**
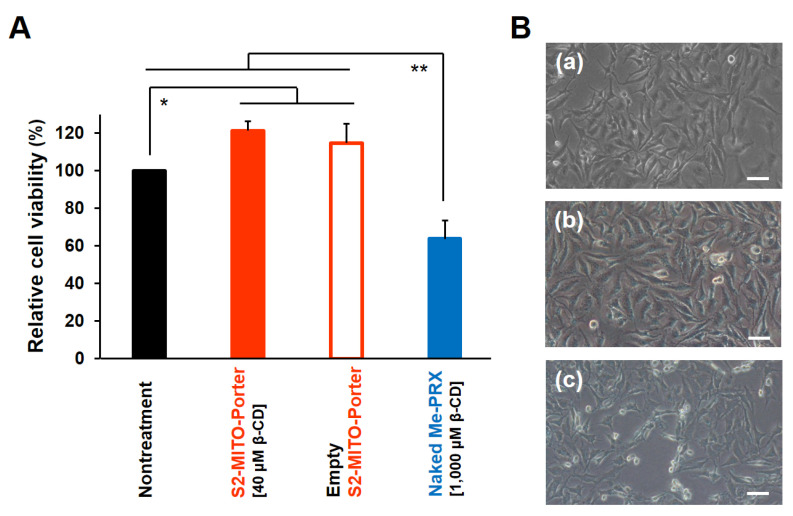
Evaluation of cell viability (**A**). At 48 h after the addition of the S2-MITO-Porter (Me-PRX) (final conc. of Me-β-CD, 40 µM), empty S2-MITO-Porter (lipid concentration equivalent to S2-MITO-Porter (Me-PRX)) and naked Me-PRX (final conc. of Me-β-CD, 1000 µM) to HeLa cells, WST-1 assays were performed to evaluate cell viability. Relative cell viability was calculated by setting the cell viability of untreated cells to 100%. Data are the mean ± S.D. (n = 3). Significant differences (** *p* < 0.01, * *p* < 0.05) were calculated by non-repeated ANOVA followed by the SNK test—observation of cell morphology (**B**). At 24 h after the addition of S2-MITO-Porter (Me-PRX) (final conc. of Me-β-CD, 40 µM) and naked Me-PRX (final conc. of Me-β-CD, 1000 µM) to HeLa cells, the cell morphology was observed using a microscope. (**a**) Nontreatment; (**b**) S2-MITO-Porter (Me-PRX) treated cells; and (**c**) naked Me-PRX treated cells. Scale bars: 30 µM.

Me-PRX induces autophagy via ER stress, ultimately leading to autophagic cell death [6]. Therefore, cell viability was assessed to ascertain whether the autophagy induced by the S2-MITO-Porter (Me-PRX) ultimately leads to cell death. At 48 h after the addition of S2-MITO-Porter (Me-PRX) to HeLa cells, a WST-1 assay was performed and cell viability relative to untreated cells was calculated (Figure 5A). The results showed that Me-PRX alone (1000 µM Me-β-CD) significantly decreased cell viability, while the S2-MITO-Porter (Me-PRX) (40 µM Me-β-CD) did not decrease cell viability, indicating that it did not induce cell death.

We also observed cell morphology at 24 h after the addition of the S2-MITO-Porter (Me-PRX) and observed a decrease in cell density and an increase in dead cells when Me-PRX alone (1000 µM Me-β-CD) was added, while in the case of the S2-MITO-Porter (Me-PRX) (40 µM Me-β-CD), no significant changes in cell morphology were observed (Figure 5B).

## 4. Discussion

In our previous study, we reported that autophagy is induced more efficiently by low concentrations of the S2-MITO-Porter (Me-PRX) than with Me-PRX alone [7]. Therefore, we hypothesized that the cellular uptake of Me-PRX via S2-MITO-Porter contributed to the change in the subcellular localization of Me-PRX. We observed the intracellular localization of Me-PRX by the S2-MITO-Porter, for each specific organelle (mitochondria, ER, and lysosomes) and compared it with the corresponding values for Me-PRX alone (Figure 2). As a result, while only negligible amounts of Me-PRX accumulated in mitochondria, the co-localization of Me-PRX and mitochondria was clearly observed when the S2-MITO-Porter was used, thus confirming that Me-PRX was delivered to mitochondria. In addition, when the S2-MITO-Porter was used, Me-PRX was observed to accumulate, not only in mitochondria and lysosomes but also, to some extent, in the ER.

In the case of Me-PRX alone, Me-PRX is localized in lysosomes, and Me-β-CD is released by degradation in an acidic environment and is then translocated to the ER [6]. In the case of the S2-MITO-Porter (Me-PRX), on the other hand, there are two possible pathways after cellular uptake. The first possibility is that the S2-MITO-Porter (Me-PRX) escapes from the endosome, and Me-PRX is delivered to the mitochondria. The second pathway involves the S2-MITO-Porter (Me-PRX) undergoing degradation in the lysosomes, and the Me-β-CD resulting from the degradation is transferred to the ER. Based on the above considerations, we conclude that it is likely that when Me-PRX is introduced into the cell via the S2-MITO-Porter, the Me-PRX is transferred to both mitochondria and the ER.

Comparison of each organelle occupancy (mitochondria, ER, and lysosomes) between the S2-MITO-Porter (Me-PRX) and Me-PRX alone showed that the level of the S2-MITO-Porter in the ER was reduced, and lysosome occupancies were comparable to that for the Me-PRX alone (Figure 2C and Figure S1). As mentioned above, when Me-PRX is introduced into the cell using S2-MITO-Porter, the Me-PRX is accumulated in both the mitochondria and the ER, which results in a lower ER occupancy than when Me-PRX alone is used. Considering the method of escape from both endosomes or lysosomes, in the case of the S2-MITO-Porter (Me-PRX), the S2 peptide modified on the lipid membrane of the S2-MITO-Porter confers membrane permeability [15,16,17], and thus, it is possible that is escapes from endosomes via the S2 peptide. On the other hand, in the case of Me-PRX alone, the Me-β-CD released in lysosomes interacts with cholesterol in the lysosomal membrane and accumulates in the ER. This difference in lysosomal occupancy suggests that the S2-MITO-Porter (Me-PRX) escapes from endosomes more efficiently than Me-PRX alone (Figure 2Cc).

When Me-PRX is delivered to mitochondria by the S2-MITO-Porter, Me-β-CD, which is generated by the degradation of Me-PRX in mitochondria, is assumed to pull out cholesterol from the mitochondrial membrane, causing mitochondrial membrane instability and a decrease in mitochondrial membrane potential. Based on previous reports, it was presumed that delivering Me-PRX to mitochondria would increase stress and that the first response to this stress would be the production of reactive oxygen species (ROS) [18]. We then hypothesized that the decrease in mitochondrial membrane potential triggers autophagy induction. However, when the mitochondrial membrane potential was evaluated when the S2-MITO-Porter (Me-PRX) was used, the mitochondrial membrane potential was confirmed to be increased. There are two possible reasons why the mitochondrial membrane potential was increased by the mitochondrial delivery of Me-PRX: first, enhanced mitochondrial metabolism by autophagy might actually improve mitochondrial function; second, autophagy-enhanced mitochondrial metabolism might increase mitochondrial mass. In order to verify these two possibilities, a more detailed evaluation of mitochondrial function using S2-MITO-Porter (Me-PRX) will be necessary. It should also be noted that there are reports that cholesterol depletion does not induce a reduction in mitochondrial membrane potential reduction [19]. Therefore, this issue needs to be investigated carefully.

The region of the ER that is in contact with mitochondria is called the mitochondria-associated membrane (MAM), where mitochondria and the ER are known to cooperate to regulate mitochondria, ER function and cellular function [20]. It has also been shown that autophagosome formation occurs in the MAM [21]. The close relationship between mitochondria and the ER via MAM suggests that reduced ER function might indirectly affect mitochondrial function. The reason for the marked decrease in mitochondrial membrane potential in the case of Me-PRX alone might be that Me-PRX induces ER stress, which indirectly injures mitochondria via MAM [22]. Therefore, Me-PRX alone might also indirectly injure mitochondria through a similar mechanism, resulting in a significant decrease in mitochondrial membrane potential.

To investigate the effect of the S2-MITO-Porter (Me-PRX) on ER function, we evaluated the expression of CHOP, a transcription factor whose expression is induced by ER stress. No change in CHOP expression was found when the S2-MITO-Porter (Me-PRX) was used (Figure 4). Hence, it was shown that when Me-PRX is introduced into cells using the S2-MITO-Porter, ER stress is not induced, and the ER is not injured, but Me-PRX is delivered to the ER. These results indicate that the mitochondrial delivery of Me-PRX is a major contributor to the induction of autophagy by the S2-MITO-Porter (Me-PRX). In addition, the activation of autophagy enhances mitochondrial metabolism in the cells, which might result in improved mitochondrial function or an increased mitochondrial mass.

## 5. Conclusions

Activation of autophagy is a mechanism that maintains cellular homeostasis by degrading and removing unwanted proteins and abnormal organelles in a cell, thereby contributing greatly to cell survival. In addition, it acts as a mechanism that leads to cell death via the activation of autophagy. The S2-MITO-Porter (Me-PRX) is responsible for the enhancement of mitochondrial metabolism by autophagy, thereby contributing to mitochondrial quality control and the maintenance of cellular function. Enhancing mitochondrial metabolism would be an effective treatment for diseases such as progressive neurodegenerative diseases and mitochondrial diseases caused by mitochondrial dysfunction and the accumulation of defective mitochondria with impaired function. To achieve this, it would be necessary to apply S2-MITO-Porter (Me-PRX) to neurons and cells derived from patients with mitochondrial diseases and to examine the effects of this strategy of artificially inducing autophagy on mitochondria in detail in order to advance this research to the treatment of diseases.

## Data Availability

The datasets used and/or analyzed during this study are available from the corresponding author upon reasonable request.

## Supplementary Materials

The following supporting information can be downloaded at: https://www.mdpi.com/article/10.3390/biom13060903/s1, Materials and Method; Figure S1: Oganelle occupancy rates were calculated based on the CLSM images. References [7,14] are cited in the Supplementary Materials.
