# Supplementary material for: Differences in the Intracellular Localization of Methylated β-Cyclodextrins-Threaded Polyrotaxanes Lead to Different Cellular States
Source: Biomolecules. 2023 May 29;13(6):903. doi: 10.3390/biom13060903 (PMC10296519; doi:10.3390/biom13060903)
Supplement: Supplementary file 1 [file biomolecules-13-00903-s001.zip › biomolecules-2400010-supplementary.pdf]

Supporting Information

Differences in the Intracellular Localization of Methylated β-Cyclodextrins-Threaded Polyrotaxanes Lead to Different Cellular States

*Yuma Yamada *, Shinnosuke Daikuhara, Atsushi Tamura, Kei Nishida, Nobuhiko Yui and Hideyoshi Harashima [1]*

Materials and Method

Synthesis of methylated β-cyclodextrin-threaded acid-labile PRX (Me-PRX): β-CD/Pluronic P103-based acid-labile PRX composed of β-cyclodextrin (β-CD, Nihon Shokuhin Kako, Tokyo, Japan) as a cyclic molecule, PEG-*b*-PPG-*b*-PEG (Pluronic P103, Adeka, Tokyo, Japan: $M_{n,PEG}$: 810 × 2, $M_{n,PPG}$: 3,250) as an axle polymer, and *N*-triphenylmethyl (*N*-Trt) groups as acid-cleavable stopper molecules was prepared as descebed in a previous report [14]. The synthesis of Me-PRX was performed as descebed in a previous report [7]. The numbers of threaded β-CDs and modified methyl groups in Me-PRX were determined by ^{1}H nuclear magnetic resonance (NMR) (Bruker Avance III 400 MHz spectrometer, Bruker BioSpin, Rheinstetten, Germany) in D_2O. The M_n of Me-PRX was calculated based on the number of threaded β-CDs and methyl groups. The number of threading β-CDs, number of modified methyl groups, and number-average molecular weight were determined to be 14.1, 234 (16.6/β-CD), and 25,000, respectively.

Synthesis of BODIPY-labeled Me-PRX: Me-PRX (50 mg, 2.0 μmol) and 1,1'-carbonyldiimidazole (Sigma-Aldrich, Milwaukee, WI, USA) (2.1 mg, 13.0 μmol) were dissolved in dehydrated DMSO (5 mL) and the solution was stirred for 18 h at room temperature under a nitrogen atmosphere. BODIPY FL EDA (Setareh Biotech, Eugene, OR, USA) (0.88 mg, 2.37 μmol) dissolved in an aliquot of dehydrated DMSO was then added to the reaction mixture, and the solution was stirred for an additional 24 hr at room temperature. The product was purified by dialysis against water (molecular weight cut-off of 3,500 Da) (FastGene) for 3

days at 4 °C. The recovered solutions were freeze-dried to yield BODIPY-Me-PRX. The absorbance of BODIPY-Me-PRX in water was measured on a V-550 UV/VIS spectrophotometer (Jasco, Tokyo, Japan), and the number of BODIPY modified on Me-PRX was determined to be 0.03.

Intracellular observation using CLSM: HeLa cells (1×10^5 cells/well) were incubated on a 3.5 cm glass-base dish (Iwaki) (5% CO_2, 37°C). After treating the cells, the mitochondrila, lysosomes and ER were stained, as described in the Experimental Section of Main text. The cells were observed by CLSM (FV10i-LIV). The cells were excited with a 473 nm light for detecting BODIPY, a 559 nm light for detecting LysoTracker Red DND-99 and CellLight ER-RFP BacMam 2.0 and a 635 nm light for detecting MitoTracker Deep Red using a LD laser. Images were obtained using a FV10i-LIV equipped with a water immersion objective lens (UPlanSApo 60x/NA. 1.2) and a diachronic mirror (DM 405/473/559/635). The fluorescence detection channels were set to the following filters: BP 490-540 (green color) for BODIPY, BP 570-620 (red color) for LysoTracker Red DND-99 and CellLight ER-RFP BacMam 2.0, and BP 660-710 (psuedo red color) for MitoTracker Deep Red.

Western-blotting: The supernatant of the cell lysate was mixed with 4×Laemmli sample buffer and incubated at 95°C for 5 min. The resulting supernatants were subjected to 12% sodium dodecyl sulfate-polyacrylamide gel electrophoresis (SDS-PAGE). The gel was prepared using a TGX FastCast acrylamide kit 12. After electrophoresis, the proteins were electroblotted onto a polyvinylidene difluoride (PVDF) membrane (0.2 μm pore size, Bio-Rad) using the Trans-Blot Turbo transfer system (Bio-Rad), and the membranes were then blocked by shaking for 1 hr at room temperature in Tris buffered saline (20 mM Tris-HCl, 137 mM NaCl, pH 7.6) with 0.1% Tween-20 (TBS-T) containing 5% nonfat dry milk. The membrane was then shaken in a 1% nonfat dry milk solution containing the primary antibodies: CHOP Rabbit Polyclonal

antibody derived from rabbit (Proteintech Group, Inc., Chicago, IL, U.S.A.) (1/1,000 dilution, 4°C, overnight) to detect the CHOP protein and a mouse anti-beta Actin monoclonal antibody derived from mice (Abcam plc, Cambridge, UK) (1/5,000 dilution, 4°C, 3 hr) to detect the β-actin (internal control). After washing the membrane three times with TBS-T, the membrane was shaken in TBS-T with 1% nonfat dry milk containing the secondary peroxidase-conjugated antibodies: Anti-IgG (H+L chain) (Rabbit) pAb-HRP (Medical and Biological Laboratories, Nagoya, Japan) (1/10,000 dilution, room temperature, 1 hr) or ECL Anti-Mouse IgG, Horseradish Peroxidase-Linked Species-Specific Whole Antibody (GE Healthcare, Little Chalfont, UK) (1/10,000 dilution, room temperature, 1 hr). After washing three times with TBS-T, the blots were developed with ImmunoStar Zeta (Wako), and immunoreactive bands were visualized using the ImageQuant LAS 4000 mini (GE Healthcare UK), and immunereactive bands were visualized using the Image J (Image Processing and Analysis in Java). We quantified the relative CHOP /β-actin protein expression levels based on the intensity of the bands corresponding to the protein.

Supplementary Figure

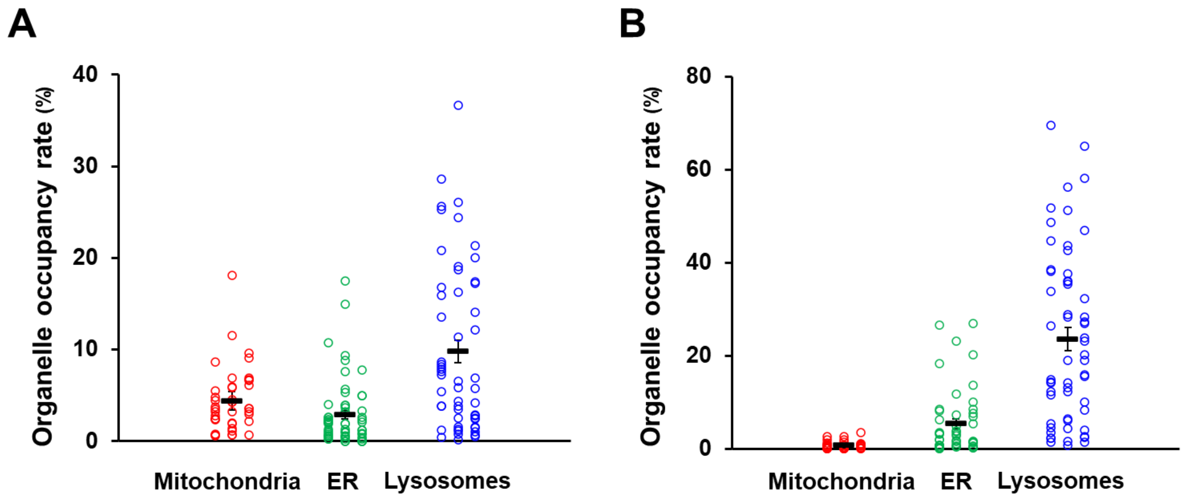

Figure S1. Oganelle occupancy rates were were calculated based on the CLSM images shown in Figures 2A and 2B, when S2-MITO-Porter (A) or naked Me-PRX (B) were used. Circles represent the values of individual cells summarized in each treatment. Bars are the mean value (n=41-58).
